# Resuscitative Endovascular Balloon Occlusion of the Aorta and Concomitant Tranexamic Acid for Cesarean Hysterectomy Complicated by Common Femoral Artery Thrombosis: A Case Report

**DOI:** 10.7759/cureus.11197

**Published:** 2020-10-27

**Authors:** Katherine Herbert, Lindsey Buchbinder, Vishwas Seshachellam, Linden Lee

**Affiliations:** 1 Department of Anesthesia and Perioperative Medicine, Medical University of South Carolina, Charleston, USA; 2 Department of Anesthesiology, McGovern Medical School University of Texas Health Science Center, Houston, USA

**Keywords:** reboa, txa, tranexamic acid, cesarean hysterectomy, thrombosis, placenta, percreta, accreta, hemorrhage

## Abstract

With increasing cesarean delivery rates, placenta accreta spectrum (PAS) disorders are occurring more frequently and represent a significant cause of peripartum hemorrhage. Different modalities have been explored to control blood loss during cesarean hysterectomies for PAS disorders, including administration of tranexamic acid (TXA) and balloon occlusion strategies. We present a case of a cesarean hysterectomy for a placenta percreta with the use of TXA and arterial balloon occlusion complicated by a lower extremity arterial thrombus requiring emergent thrombectomy. The outcome of this case suggests using caution with concomitant use of TXA and arterial balloon occlusion.

## Introduction

Maternal hemorrhage continues to be a leading cause of maternal morbidity and mortality across the world. Placenta accreta spectrum (PAS) disorders are a major contributor to maternal hemorrhage and are occurring more frequently as cesarean delivery rates are increasing [1]. PAS disorders are defined by pathologic placental adherence and include placenta accreta, placenta increta, and placenta percreta. Placenta accreta involves attachment of the placental chorionic villi to the myometrium, placenta increta involves invasion into the myometrium, and placenta percreta involves penetration through the serosa and potentially into adjacent pelvic organs [2].

Conventional treatment of peripartum hemorrhage includes uterine massage, administration of uterotonics, fluid replacement, and blood product transfusion [2]. However, in the presence of an abnormally adherent placenta and neovascularization, these techniques may not be effective, requiring escalation to other modalities. These modalities include additional pharmacological agents and blood products, uterine artery embolization, arterial balloon occlusion, or hysterectomy. Balloon occlusion of the bilateral internal iliac arteries and the abdominal aorta has been used to reduce blood loss [3-5].

Fibrinolysis can play a significant role in peripartum and traumatic hemorrhage [6]. This finding led to the Clinical Randomization of an Antifibrinolytic in Significant Hemorrhage 2 (CRASH-2) and World Maternal Antifibrinolytic (WOMAN) trials which demonstrated that the antifibrinolytic agent tranexamic acid (TXA) reduced mortality from traumatic and peripartum hemorrhage [7,8]. These studies also suggested that TXA does not significantly increase the risk of embolic complications. 

Due to the risk of massive hemorrhage and the resulting morbidity with PAS disorders, multidisciplinary collaboration is necessary to optimize patient outcomes. In our case, trauma surgery was included for placement of Resuscitative Endovascular Balloon Occlusion of Aorta (REBOA, Prytime Medical, Boerne, TX), a minimally invasive device that can temporarily occlude the aorta to control bleeding and improve afterload in patients with traumatic hemorrhagic shock [9].

We present a patient who underwent cesarean hysterectomy for placenta percreta with prophylactic REBOA use and TXA administration complicated by right common femoral and iliac artery thrombi requiring emergent thrombectomy. A written Health Insurance Portability Accountability Act (HIPAA) authorization to use/disclose protected health information was obtained.

## Case presentation

**Figure 1:**
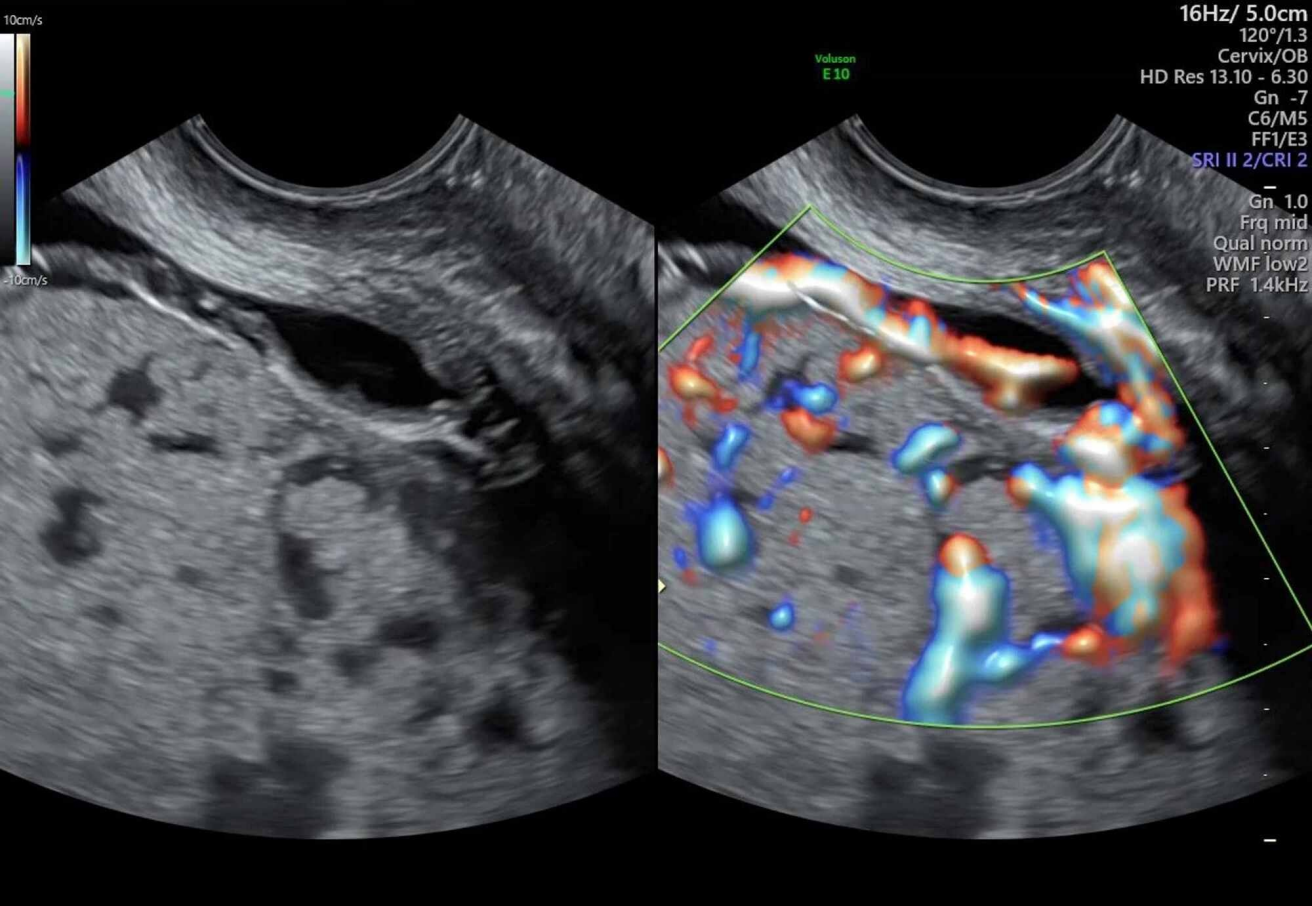
Transabdominal ultrasound image displaying engorged vessels extending along the junction of the bladder with the placenta and uterus. The vessels appear to extend into the bladder, raising suspicion for placenta percreta

A 37-year-old G2P1 female with a history of one prior cesarean delivery presented at 25 weeks gestational age for vaginal bleeding. She was noted to have a placenta previa and suspected placenta percreta on ultrasound (Figure 1). MRI results were concerning for placental invasion into the bladder and surrounding vascular structures. Due to the severity of invasion, neovascularization, and the high likelihood of acute hemorrhage that would be challenging to control, a multidisciplinary team planned to perform a cesarean hysterectomy at 32 weeks gestational age with prophylactic REBOA placement.

Prior to surgery, a massive transfusion protocol pack, rapid infuser, and intraoperative cell salvage device were on standby in the operating room. A right radial arterial line was placed prior to induction of general anesthesia, and a right internal jugular vein central line was placed atraumatically. Cystoscopy was not suggestive of placenta invasion through the entire bladder wall; however, it did reveal mass effect with vascularity just below the bladder mucosa. Femoral arterial cannulation for REBOA placement was challenging and attempts were performed on both common femoral arteries prior to the successful placement of a 7 French Cordis sheath in the patient’s right common femoral artery. Significant hemorrhage occurred from the hysterotomy site after delivery of the fetus requiring REBOA inflation for three minutes and blood transfusion via rapid infuser. There was found to be partial invasion of the placenta into the bladder, which resulted in significant hemorrhage, subsequent hysterectomy, and partial cystectomy during which the REBOA was re-inflated for 30 minutes. Bleeding from multiple sites was noted upon examination of the uterus, and TXA 1 gram was administered due to ongoing hemorrhage. Thromboelastography revealed normal coagulation parameters. The hysterectomy was completed, and surgical hemostasis achieved. The patient's mean arterial pressure was maintained at greater than 60 mmHg throughout the case. The total estimated blood loss was 4000 ml, and the patient received a total of 5 units of cell saver, 3 units of packed red blood cells, and 3 units of fresh frozen plasma. Bilateral quadratus lumborum blocks were performed for postoperative analgesia prior to extubation in the setting of a positive cuff leak test.

After an uneventful post-anesthesia care unit (PACU) stay, the patient was transported to the ICU for serial neurovascular checks and the sheath of the REBOA was removed. Later that evening, decreased pulses were identified in her right lower extremity, prompting an emergent angiogram and thrombectomy of the right external iliac and common femoral arteries under general anesthesia. The remainder of her hospital stay was otherwise uneventful. At the time of discharge, the patient required a walker for ambulation due to right lower extremity pain with weight-bearing. At four weeks post-operatively, she was able to ambulate without assistance or pain.

## Discussion

Peripartum hemorrhage from abnormal placental implantation is a significant contributor to maternal morbidity and mortality. PAS disorders include a range of pathologically adherent placentas from placenta accreta to the more invasive cases of placenta increta and placenta percreta. The incidence of PAS disorders has continued to rise over the last several decades, with sources attributing this change due to an increase in the primary risk factor - prior cesarean delivery [1]. Other risk factors for PAS include placenta previa, advanced maternal age, multiparity, prior uterine surgeries, and Asherman syndrome [1]. 

Peripartum hemorrhage is defined as greater than 500 mL blood loss for vaginal delivery and greater than 1000 mL for cesarean delivery. However, with PAS disorders, the average blood loss can range from 3,000 mL to 5,000 mL [4]. Bleeding can be challenging to control in these patients due to difficult visualization, neovascularization, and adherent or altered anatomy. Massive hemorrhage can lead to hypovolemia, hypotension, coagulopathy, and transfusion of large amounts of blood products, which may result in transfusion reactions, airway or peripheral edema, electrolyte abnormalities, and the development of antibodies. Our hope was to minimize the likelihood of these adverse outcomes in our patient's case by utilizing techniques to minimize hemorrhage.

REBOA has proven to be effective in controlling massive traumatic hemorrhage; however, further investigation is necessary to define its utility in the obstetric population. A recent systematic review examined peripartum blood loss in women with abnormal placentation that received prophylactic REBOA placement [4]. The median blood loss was 1500 mL with two-thirds of the study group having less than 3000mL blood loss, suggesting that prophylactic REBOA placement may have helped to reduce overall blood loss in these patients [4]. In our case, the use of REBOA limited severe blood loss allowing us to avoid massive transfusion and to extubate our patient safely at the end of the case.

The use of REBOA does not come without risk. One study in which REBOA was used to control severe postpartum hemorrhage in 36 patients demonstrated that five patients developed local thrombus formation of the iliac or femoral artery [3]. Pregnancy is known to be a hypercoagulable state with an elevated risk of venous thromboembolism until 12 weeks postpartum [10]. Changes include increased levels of factor VII, VIII, X, von Willebrand factor, and fibrinogen, decreased protein S concentrations, and impaired fibrinolysis [10]. While these changes in the coagulation profile are beneficial for limiting blood loss in normal patients during the immediate postpartum period, thromboembolic complications may develop especially in patients with multiple risk factors. 

TXA is an antifibrinolytic agent and has been shown to reduce the incidence of hemorrhage in liver, cardiac, orthopedic, and gynecologic surgeries [11]. Bleeding is reduced by the inhibition of plasmin in the breakdown of fibrinogen and fibrin [6]. The WOMAN trial demonstrated in a large multicenter study that the administration of TXA reduced mortality from peripartum hemorrhage [7]. The CRASH-2 trial showed that early administration of TXA to trauma patients reduced the risk of death from hemorrhage with no increase in fatal or non-fatal vascular occlusive events. However, the group mentions that due to the low precision of their data, it must be considered that there is some possibility of increased risk of thrombosis [8]. Hajmurad et al. describe a case similar to ours in which a patient undergoing cesarean hysterectomy for placenta percreta received bilateral internal iliac artery balloons and intraoperative TXA. This patient developed bilateral common femoral artery thrombi requiring embolectomy [12]. While REBOA and TXA appear to show promise in the treatment of peripartum hemorrhage, the findings from our case in combination with those from Hajmurad et al. would suggest using caution when utilizing these two therapies together in hypercoagulable patients. Femoral arterial cannulation for REBOA placement in our patient also was challenging and required several attempts. These repeated attempts may have resulted in increased endothelial injury and possibly an increased likelihood of thrombus development.

This article was previously presented as a poster at the American Society of Anesthesiologists Annual meeting on October 22, 2019.

## Conclusions

We suggest reserving the use of REBOA in patients with PAS disorders to severe cases in which bleeding is anticipated to be challenging to control. An experienced physician should be the one to obtain vascular access to minimize the number of attempts and endothelial injury. If REBOA is utilized, it may be worthwhile to consider administering TXA only if fibrinolysis is identified on point-of-care coagulation studies. Further research is necessary to determine the best applications of REBOA and TXA to limit blood loss and complications in patients with PAS disorders.
